# Relative risk of primary bloodstream infection in patients with mechanical circulatory support devices

**DOI:** 10.1017/ash.2023.216

**Published:** 2023-09-29

**Authors:** Rachel Wolansky, Patrick Burke, Ryan Miller, Thomas Fraser

## Abstract

**Background:** Patients requiring mechanical circulatory support (MCS) during episodes of cardiogenic shock are at risk for hospital-acquired bloodstream infection (HABSI). Clinically MCS devices include extracorporeal membrane oxygenation (ECMO) devices, durable and temporary left ventricular-assist devices (VADs), and intra-aortic balloon pumps (IABPs). However, the MCS exclusion to the NHSN central-line–associated bloodstream infection (CLABSI) surveillance rules in 2018 did not include IABP as a qualifying device. We have described utilization and incidence of primary HABSI (pHABSI) in our patients requiring MCS. **Methods:** The setting for this study was 9 cardiothoracic and heart failure intensive care units with 131 total beds at the Cleveland Clinic Main Campus. Surveillance for HABSI to include determination of CLABSI was performed prospectively. MCS-associated pHABSI were patients who had ECMO, LVAD, or IABP present for >2 calendar days with device in place on the date of infection or removed the day before. A patient with 2 device types at time of infection was counted as a pHABSI for both groups. Patient, device, and MCS days were extracted from an electronic database. Non-MCS patient days were calculated as the difference between total patient days and total MCS days. The incidence of ECMO-, VAD-, and IABP-associated pHABSI were compared to each other and to non–MCS-associated pHABSI using OpenEpi version 3.01 software. **Results:** Surveillance results are shown in Table 1. During the observation period, there were 221 pHABSIs and 139,013 patient days. Moreover, 67 pHABSIs were associated with an MCS device over 17,044 total MCS days: 43 ECMO days, 18 VAD days, and 13 IABP days. Also, 9 patients had >1 type of eligible device and 7 (39%) of the IABP-associated pHABSIs were CLABSIs.

The cumulative incidences of pHABSI associated with ECMO, VAD, and IABP were 5.68, 4.59, and 2.34 per 1,000 MCS days, respectively. The incidence of IABP pHABSI was not significantly different from VAD pHABSI (*P* = .06), but it was different from ECMO pHABSI (*P* < .01). The pHABSI rate for non-MCS days was 1.26 per 1,000 patient days. **Conclusions:** In our patients requiring MCS, the risk of pHABSI associated with IABP was significantly greater than in patients without MCS and was similar to patients with VAD. MCS of all types should be considered a risk for HABSI in patients with cardiogenic shock beyond the presence of a central line. Protocols to further prevent HABSI morbidity in IABP patients are needed.

**Figure:**
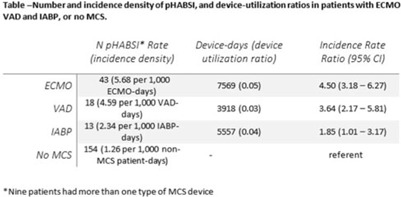

**Figure:**
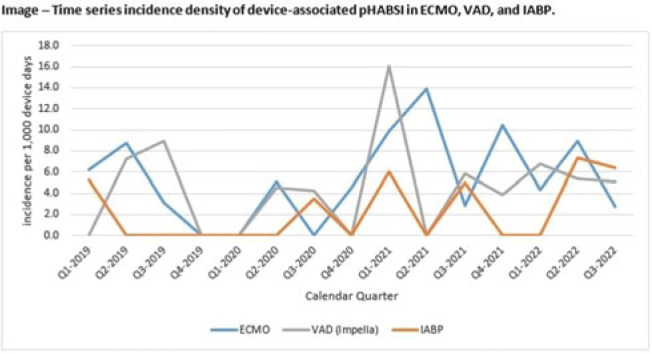

**Disclosure:** None

